# Composite Nanoarchitectonics of Electrospun Piezoelectric PVDF/AgNPs for Biomedical Applications, Including Breast Cancer Treatment

**DOI:** 10.3390/ma17153872

**Published:** 2024-08-05

**Authors:** Strahinja Milenković, Katarina Virijević, Fatima Živić, Ivana Radojević, Nenad Grujović

**Affiliations:** 1 Institute for Information Technologies, University of Kragujevac, 34000 Kragujevac, Serbia; strahinja.milenkovic@kg.ac.rs (S.M.); katarina.virijevic@uni.kg.ac.rs (K.V.); 2 Faculty of Engineering, University of Kragujevac, 34000 Kragujevac, Serbia; gruja@kg.ac.rs; 3 Department of Biology and Ecology, Faculty of Natural Sciences, University of Kragujevac, 34000 Kragujevac, Serbia; ivana.radojevic@pmf.kg.ac.rs

**Keywords:** PVDF/AgNPs composite nanofibers, silver nanoparticles (AgNPs), piezoelectricity, electrospinning, cytotoxicity, antibacterial activity, breast cancer cells

## Abstract

This study focused on preparing composite nanomats by incorporating silver nanoparticles (AgNPs) in polyvinylidene fluoride (PVDF) nanofibers through the electrospinning process. A short review of piezoelectric PVDF-related research is presented. PVDF is known for its biocompatibility and piezoelectric properties. Since electrical signals in biological tissues have been shown to be relevant for therapeutic applications, the influence of the addition of AgNPs to PVDF on its piezoelectricity is studied, due to the ability of AgNPs to increase the piezoelectric signal, along with providing antibacterial properties. The prepared samples were characterized by scanning electron microscopy, energy-dispersive X-ray spectroscopy, and Fourier transform infrared spectroscopy. In addition, the biological activity of composites was examined using a cytotoxicity assay and an assessment of the antibacterial activity. The obtained results show that the incorporation of AgNPs into PVDF nanofibers further enhances the piezoelectricity (crystalline β-phase fraction), already improved by the electrospinning process, compared to solution-casted samples, but only with a AgNPs/PVDF concentration of up to 0.3%; a further increase in the nanoparticles led to a β-phase reduction. The cytotoxicity assay showed a promising effect of PVDF/AgNPs nanofibers on the MDA-MB-231 breast cancer cell line, following the non-toxicity displayed in regard to the healthy MRC-5 cell line. The antibacterial effect of PVDF/AgNPs nanofibers showed promising antibacterial activity against *Pseudomonas aeruginosa* and *Staphylococcus aureus*, as a result of the Ag content. The anticancer activity, combined with the electrical properties of nanofibers, presents new possibilities for smart, multifunctional materials for cancer treatment development.

## 1. Introduction

Energy-harvesting and energy-conversion lead-free materials have gained significant attention [1,2,3,4], because they are environmentally friendly solutions, while being promising materials to solve challenges related to a range of applications where traditional electronic materials are hard to use, such as in relation to power devices for medical implants [5], self-powered implantable and wearable devices [6], wearable nanogenerators [7,8,9], self-healing stretchable electronics [10,11], human motion monitoring [12], neuroregeneration [13,14], tissue engineering [15,16], faster wound healing [17,18], actuation in soft robotics [19], force sensors [20], advanced water purification applications [21], self-cleaning nanofiltration membranes in wastewater treatment [22], devices for the control of the energy release [23], environmental monitoring sensors [24], devices for vibration control or smart construction materials [25], coatings that can function as energy harvesting device that respond to UV [26], and a wide range of different electromechanical devices [27].

Medical implants that can perform diagnostics and that can be used for control and treatment through the use of piezoelectric materials can be designed to operate without an external power source, thus enabling wireless and batteryless devices that can be implanted, for example, to monitor continuous physiological signals in real time [5,28]. The development of biosensors that can perform continuous health monitoring is a very significant area of research for biomedical implants [29,30], but that research can also greatly contribute to a wide range of small, self-sustainable industrial electronics for novel Internet of Things (IoT) applications [31], or for use in advanced medical devices [32].

Poly(vinylidene fluoride) (PVDF), a piezoelectric polymer, has been investigated for its use in sensing and actuating applications for a long time [33]. The piezoelectric properties of PVDF make it ideal for converting mechanical stress into electrical energy, which is useful in a variety of sensing and energy-harvesting applications [34,35]. From 1969 onwards, polar polymers have been studied that exhibit piezo-, pyro-, and ferroelectricity, including PVDF, copolymers of vinylidene fluoride and trifluoroethylene, aromatic and aliphatic polyureas, polyurethane, vinyl cyanide, vinyl acetate, and nylon [27]. PVDF can contain different crystalline polymorphs (α, β, γ, δ, and ε phases), whereas only the β phase exhibits piezo-, pyro-, and ferroelectric properties [36,37].

The fabrication of PVDF with a dominant β phase is challenging, where mechanical stretching is one of the routes used to increase the β-phase content, since it realizes the transformation of the α phase to the β phase [38,39,40]. Transformation of the α phase to the β phase is significantly influenced by the temperature and stretching ratio, whereas these material modifications further influence the degree of crystallinity and the microstructure, thus producing different final macroscopic material responses [38]. The PVDF polymerization process parameters can also provide tailoring of the specific phase contents [41]. Different fabrication routes have been studied, including electrospinning, solution casting, spin coating, the hot-press method, self-poling, melt blending, soft lithography, and additive manufacturing (3D printing and solvent evaporation-assisted 3D printing) [42,43].

Electrospinning technology performs mechanical stretching of the liquid solution droplet within the electric field to form long nanofibers and, in the case of a PVDF-based solution, that stretching is favorable for creating the β phase in the final PVDF nanofibers [44,45]. Electrospinning process parameters have a significant influence on the resulting fraction of the β phase, the nanofiber morphology, and the piezoelectric output [46].

Nanocomposites of PVDF reinforced with different nanoparticles have been studied to increase the resulting piezoelectric response. A graphene oxide (rGO)-Ag/PVDF nanocomposite was studied for use in a self-poled piezoelectric nanogenerator [47] and Ag-CuO/rGO-PVDF nanocomposite was studied, with an improved polar β phase for polymer batteries [48]. The development of piezoelectric materials drives the further development of advanced magnetoelectric biomaterials for biomedical applications, such as advanced sensors, tissue engineering, anticancer treatments, drug delivery systems, and micro-devices that provide antimicrobial functions [49,50,51].

Silver nitrate (AgNO_3_) is a well-known antimicrobial agent that has been used for centuries to inhibit the growth of microbes. It has broad-spectrum antimicrobial and anticancer effects due to the release of silver ions (Ag^+^) that disrupt the membranes of cancer cells and the metabolism of microorganisms. Silver nanoparticles (AgNPs) have been studied as fillers in the PVDF matrix and have been proven to enhance the piezoelectric output [52]. The combination of PVDF and AgNPs results in a multifunctional nanocomposite that takes advantage of PVDF’s piezoelectricity and the biological efficacy of AgNPs. The composite PVDF nanofibers with AgNPs exhibit improved thermal and mechanical properties and better biological activity against a variety of pathogens, due to the oligodynamic effect of silver [53,54].

Recent studies have investigated the cytotoxic effects of AgNPs on cancer cells [55,56] and indicated a potential pathway for anticancer treatments through the use of PVDF/AgNPs nanofibers. It has been suggested that AgNPs can initiate apoptosis in malignant cells by producing reactive oxygen species and impairing mitochondrial function [57]. Such anticancer activity of the material, combined with its piezoelectrical properties, opens up new possibilities for the development of smart, multifunctional materials for cancer treatment where PVDF nanofibers with AgNPs are of particular interest in this context.

The objective of our research was to synthesize and investigate the properties and potential applications of PVDF/AgNPs nanofibers focusing on their piezoelectric capabilities, antimicrobial efficacy, and anticancer effects. A short review of the piezoelectric PVDF-related research has been also presented. We studied the process parameters of electrospinning (temperature, solvents, percentage of PVDF) for the fabrication of nanofibers and we further performed their characterization. PVDF/AgNPs fiber mats (or nanomats) were characterized by SEM, EDS microscopy, and FTIR spectroscopy. We tested the antibacterial properties and biological and anticancer activity against breast cancer cells. To the best of our knowledge, this is the first such study related to the breast cancer cells.

## 2. Materials and Methods

Poly(vinylidene fluoride) (PVDF, Mw~180.00 by GPC) was purchased from Sigma Aldrich (St. Louis, MO, USA). Acetone (Ac, ≥99.5%) was purchased from Honeywell (Charlotte, NC, USA), and dimethylformamide (DMF, ≥99.5%) and silver nitrate (AgNO_3_, ≥99.9%) were purchased from Fisher Chemical (Waltham, MA, USA). Dulbecco’s Eagle Medium (DMEM), 1% antibiotic agent penicillin/streptomycin, 10% Fetal Bovine Serum (FBS), and 0.25% trypsin-EDTA were provided from Sigma Aldrich (St. Louis, MO, USA). MTT (3-[4,5-dimethylthiazol-2-yl]-2,5-diphenyltetrazolium bromide) was purchased from Acros Organics (Geel, Beligum). In this study, all chemicals were applied without further purification.

### 2.1. Fabrication of the PVDF Nanofibers

Seven different chemical compositions of PVDF were prepared and tested. The most conductive solution was the one with 21% of PVDF and in a combination of Ac and DMF co-solvents at a 1:3 ratio. The presence of the DMF solvent plays a decisive role in reducing the concentration of Ag+ ions. This reduction process facilitates the formation of metallic silver (Ag0), followed by the aggregation of oligomeric groups. Over time, these groups combine to form colloidal silver metal particles [58]. Previous research has shown that DMF is a suitable reducing agent for Ag+ ions, as proven by the formation of AgNPs in DMF [59]. Also, the interaction between DMF and PVDF results in a –CH2/–CF2 dipole forming, and increases the content of the β phase [60]. The best solution and electrospinning parameters are presented in Table 1.

The prepared solution with 21% of PVDF was stirred at 80 °C for 3 h to achieve a homogeneous blend. Varying amounts of AgNO_3_ (0.1%, 0.3%, and 0.5% *w*/*w*) were added to the PVDF solution, followed by a 12 h stirring period. After that, the mixture was dispersed using an ultrasonicator and cooled to 28 °C. The solution became grey, indicating that AgNPs were formed.

Subsequently, the prepared solutions were loaded into a 5 mL syringe with attached 18-gauge needle. Electrospinning was conducted by applying a voltage of 30 kV, and maintaining a flow rate of 0.5 mL/h, with a distance needle collector of 15 cm. During the electrospinning process, optimal conditions were maintained at 45% humidity and a temperature of 30 °C. After electrospinning, the nanofibers were carefully collected on the aluminum foil and stored in a dark environment at room temperature to facilitate the removal of the residual solvent content.

**Figure 1:**
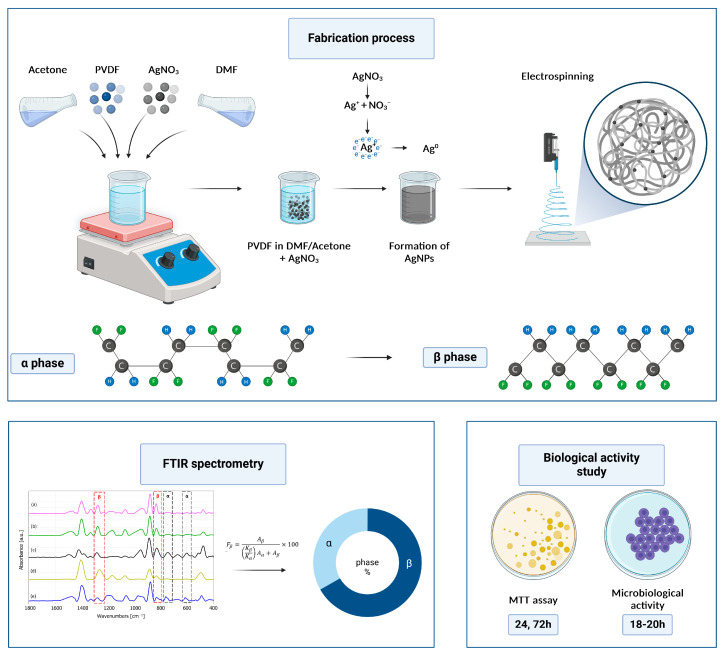
PVDF nanofibers fabrication and evaluation process.

The synthesis process of the PVDF/AgNPs nanofibers and the concept of characterization is presented in Figure 1.

### 2.2. Scaffold Characterization and Morphology

The morphology and diameters of the obtained nanofibers were examined with an inverted microscope (Delta Optical Genetic PRO microscope, Warsaw, Poland) and a scanning electron microscope (SEM) (FEI Scios2 Dual Beam System, Hillsboro, OR, USA) at the Institute of Nuclear Sciences Vinča, University of Belgrade, Serbia. Image J software (version 1.48) was used to analyze the fiber diameters. The average fiber diameter was calculated using 150 measurements from three different micrographs (50 measurements per micrograph). Furthermore, the collected data were systematically processed and analyzed using the SPSS software (ver. 17) to determine the fiber diameter distribution. The resulting histograms prominently displayed the average fiber diameter and the standard deviation, thus enabling a comprehensive understanding and interpretation.

### 2.3. SEM and EDS Analysis

Square samples of 2 cm × 2 cm sizes were cut and coated with gold for 30 s, as a preparation procedure for the SEM analysis. The coated samples were carefully placed in an SEM microscope, at 10 kV. Furthermore, energy-dispersive spectroscopy (EDS) was used for the elemental quantification to detect the presence of Ag-loaded nanofibers.

### 2.4. FTIR Spectroscopy Analysis

The presence of crystalline phases in the electrospun PVDF samples was determined by transmission infrared spectroscopy (portable FTIR/FT-NIR spectrometer Interspec 301-X, Toravere, Estonia) at the range between 400 cm^−1^ and 1600 cm^−1^ with a resolution of 4 cm^−1^. The film sample was prepared through the solution evaporation method for the FTIR analysis to determine whether the β phase was present in PVDF. The PVDF solution was cast on the substrate and left overnight for the solvent to evaporate [61].

### 2.5. Biological Activity of the PVDF/Ag Electrospun Nanofibers

The biological activity of the PVDF and PVDF/AgNPs electrospun nanofibers was studied through the cell viability, cytotoxicity, and microbiological activity.

#### 2.5.1. Cytotoxicity Assay (In Vitro Cell Culture Study)

The cell viability, growth, and cytotoxicity of the PVDF and PVDF/AgNPs electrospun nanofibers (0.1%, 0.3%, and 0.5% AgNPs) were studied by an MTT standardized protocol [62,63]. The MTT assay takes advantage of the activity of dehydrogenase enzymes produced by the metabolically active cell mitochondria, which catalyze the conversion of the yellow tetrazolium salt to the purple formazan crystals. The resulting purple color inversely correlates with the number of viable cells, resulting in a precise indicator of the cell viability. The PVDF and PVDF/Ag electrospun nanofibers were cut into 5 × 5 mm round samples, placed into the 96-well plates, and sterilized by UV irradiation, followed by washing in 70% ethanol. After that, DMEM was added to the bottom of the 96-well and incubated for 24 h for better cell adhesion onto the nanofibers. The next step was DMEM aspiration, and the scaffolds were seeded with MRC-5 (healthy fibroblasts from pleura lung) cells at a density of 2.5 × 10^4^ cells/100 µL. The positive control wells were seeded with cells without the scaffolds.

Afterwards, the supernatant was removed and 125 mL of MTT solution (5 mg/mL) was added into each well and incubated at 37 °C and 5% CO_2_ for 3 h. After incubation, the MTT solutions were discarded, and 150 µL of DMSO was added to dissolve the formazan crystals. The absorbance was measured at 490 nm using a microplate. Cell viability was determined after 24 h and 72 h. The optical density of the purple formazan solution that indicated cell viability, was quantified using a Multiskan SkyHigh Microplate Spectrophotometer (ThermoFisher Scientific Inc., Waltham, MA, USA.) at a wavelength of 492 nm. Each sample was analyzed three times, and data are expressed as the mean value ± standard error.

#### 2.5.2. Microbiological Assessment

The antibacterial activity of the PVDF nanofibers with AgNPs (0.1, 0.3, and 0.5%) was tested against the two standard strains of bacteria. The experiment involved one Gram-positive bacteria (*Staphylococcus aureus*, ATCC 25923) and one Gram-negative bacteria (*Pseudomonas aeruginosa*, ATCC 27853). Gentamicin susceptibility test discs of 10 μg (Bioanalyse, Ankara, Turkey) were used as a positive control.

The bacterial cultures were cultivated on a nutrient agar before the experiment. The incubation period lasted for 18–20 h, at 37 °C temperature. The bacterial suspensions were prepared by the direct colony method. The suspension turbidity was adjusted using a densitometer (DEN-1, BioSan, Riga, Latvia), McFarland 0.5, corresponding to 10^8^ CFU/mL. The bacterial suspensions were prepared immediately before the experiment, as it is recommended to use them approximately within the timeframe of 15 min after the preparation [64].

The sensitivity of bacteria to PVDF nanofibers with different concentrations of AgNPs was tested by the in vitro disk diffusion method. The disk diffusion test was performed in a Petri dish on the Mueller–Hinton agar (25 mL of medium per plate). PVDF nanofibers with AgNPs and gentamicin susceptibility test discs were placed on the surface of the medium (3 identical nanofibers on 1 plate), on which a pure bacterial suspension with 1–2 × 10^8^ CFU/mL was cultivated. After incubation (16–24 h), the inhibition zone diameter was measured. All zones of inhibition were calculated three times.

#### 2.5.3. Statistical Analysis

All data are presented as the mean value ± standard error (SE). A one-way ANOVA test was used to determine statistical significance. The magnitude of correlations between variables was calculated using the SPSS statistical software package (Chicago, IL, USA) (SPSS for Windows, ver.17, 2008).

## 3. Results

### 3.1. Characterization and Morphology of the PVDF and PVDF/AgNPs Nanofibers

**Figure 2:**
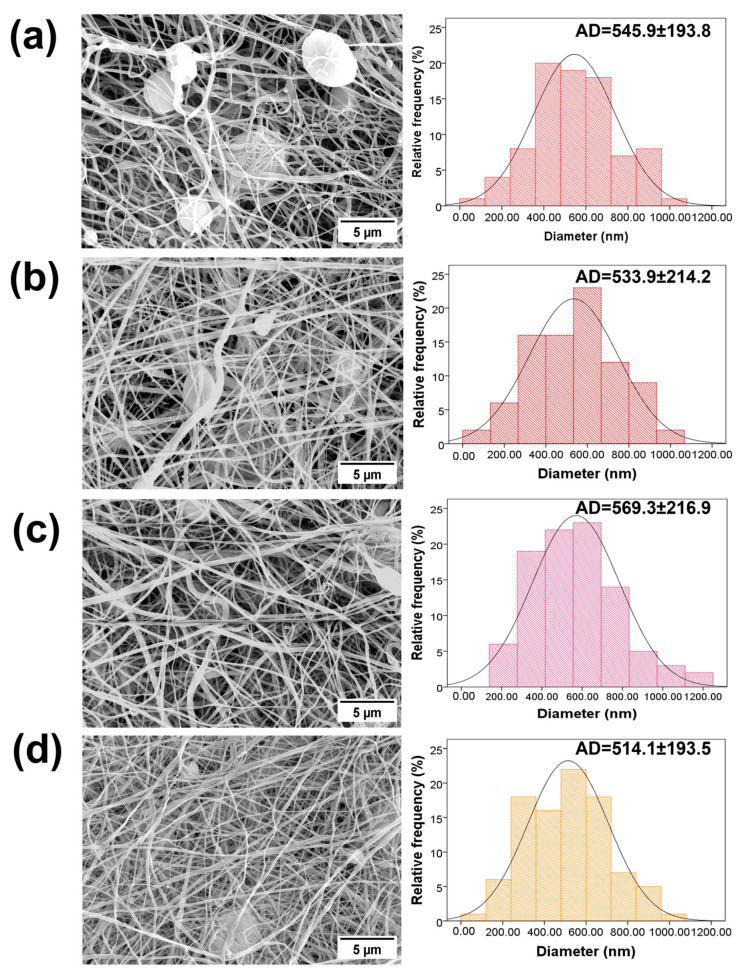
SEM micrographs of the PVDF and PVDF/AgNPs with different concentrations of incorporated AgNPs, with diagrams of the diameter distribution: (**a**) pure PVDF nanofibers (**b**) PVDF/0.1% AgNPs (**c**) PVDF/0.3% AgNPs, and (**d**) PVDF/0.5% AgNPs nanofibers.

**Figure 3:**
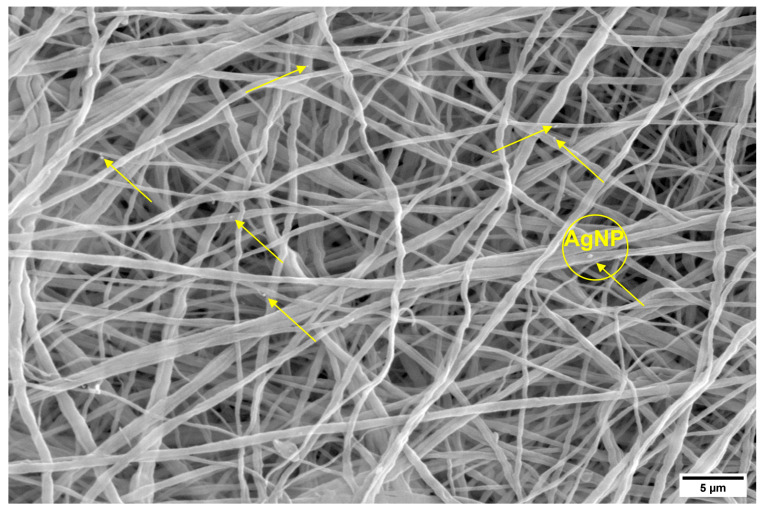
Silver nanoparticles (AgNPs) distributed throughout the PVDF nanofibers, as denoted by the arrows in the image.

SEM images and the diameter distributions of PVDF and PVDF/AgNPs nanofibers are presented in Figure 2. The presence of AgNPs in PVDF nanofibers shows visible differences in morphology and average diameters. Silver nanoparticles are not visible in Figure 2, due to a very low concentration of initially added AgNO_3_. The higher concentrations of AgNO_3_ (1%, 3%, and 5%) during this study showed a strong cytotoxicity effect with prominent killing of both the healthy MRC-5 and MDA-MB-231 cells. Randomly distributed AgNPs can be observed in Figure 3. The size of AgNPs is calculated by the ImageJ software. The average value size is 19.8 nm. According to the literature data, the modified membranes (for example PA/PVDF) that contain silver nanoparticles (from 20 nm to 50 nm) showed antibacterial properties against Gram-negative bacteria in the form of 3–4 mm inhibitory zones [65].

Specifically, pure PVDF fibers exhibit an average diameter of 545.9 ± 193.8 nm, whereas the diameters of the PVDF/AgNPs nanofibers have diameters from 514.1 ± 193.5 to 569.3 ± 216.9 nm, depending on the concentration of AgNPs. An increase in the nanofiber diameter, together with the presence of beads is noted in the pure PVDF sample (average diameter of 545.9 ± 193.8 nm). Upon the incorporation of 0.1% AgNPs into the PVDF, a reduction in the bead formation was observed, although it is still present in the structure, together with the decrease in the fiber diameter to 533.9 ± 214.2 nm. It should be noted that the addition of 0.5% AgNPs resulted in almost full formation of beads in the PVDF morphology, together with a further decrease in the fiber diameter to 514.1 ± 193.5 nm. However, in the case of the PVDF/0.3% AgNPs nanofibers, an unexpected increase in the fiber diameter to 569.3 ± 216.9 nm is noted, which is a higher diameter than in the case of the pure PVDF fibers. The SEM observation showed that incorporated silver did not affect the morphology of the PVDF nanomats and increased the structural integrity of the fibers.

#### 3.1.1. EDS Analysis

**Figure 4:**
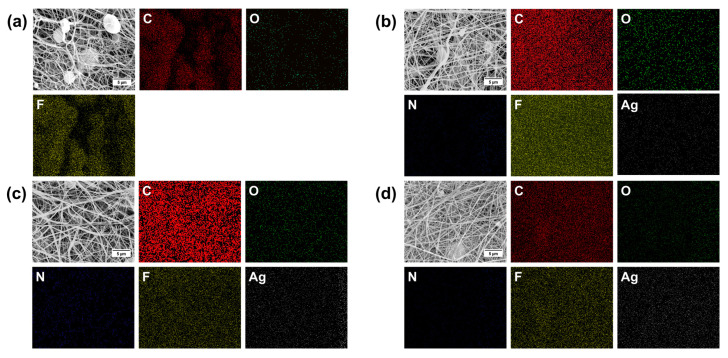
EDS analysis of (**a**) pure PVDF (**b**) PVDF/0.1% AgNPs (**c**) PVDF/0.3% AgNPs, and (**d**) PVDF/0.5% AgNPs nanofibers related to the distribution of C (red), N (dark blue), O (green), F (yellow), and Ag (white). The scale bar is 5 µm.

**Figure 5:**
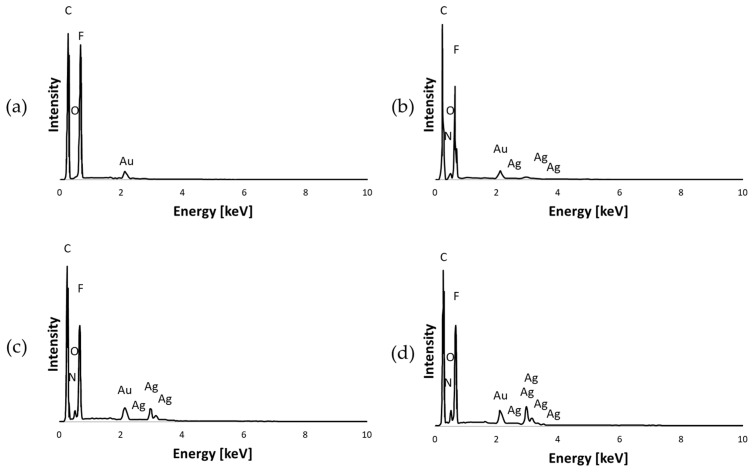
EDS diagrams of elemental distribution of specified sample: (**a**) pure PVDF (**b**) PVDF/0.1% AgNPs (**c**) PVDF/0.3% AgNPs, and (**d**) PVDF/0.5% AgNPs.

The elemental composition and presence of silver in the PVDF nanofibers were confirmed by energy-dispersive spectroscopy (EDS). Figure 4 shows the EDS mapping of the pure PVDF, PVDF/0.1% AgNPs, PVDF/0.3% AgNPs, and PVDF/0.5% AgNPs nanofibers. The presence of carbon (C), oxygen (O), nitrogen (N), and fluor (F), as well as silver (Ag) atoms can be seen. The EDS spectrum (Figure 5) demonstrates the existence and homogeneous distribution of Ag, thus indicating the structural integrity and successful binding of the silver nanoparticles to the PVDF nanofibers.

#### 3.1.2. FTIR Spectroscopy Analysis

**Figure 6:**
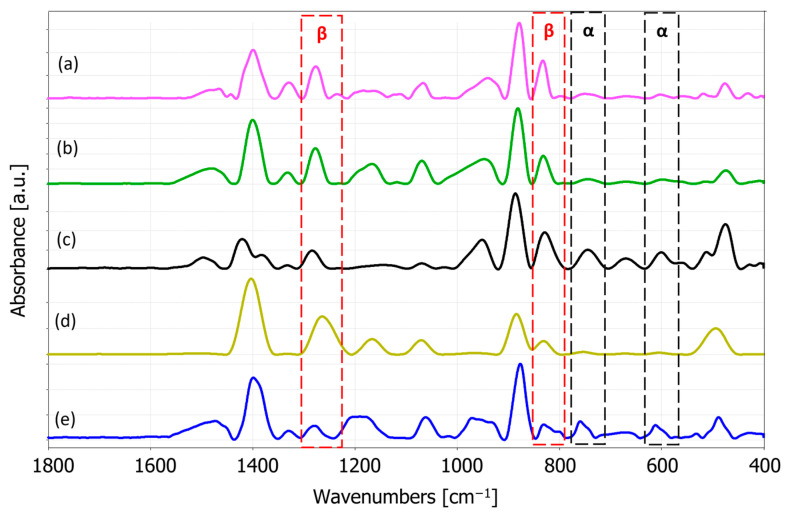
FTIR absorbance spectra of the PVDF: (a) PVDF/0.1% AgNPs, (b) PVDF/0.3% AgNPs, (c) PVDF/0.5% AgNPs, (d) electrospun pure PVDF fibers, and (e) solution-casted PVDF film.

The FTIR spectra of pure PVDF and PVDF/Ag nanofibers are shown in Figure 6. The absorption peak around 1400 cm^−1^ is attributed to the PVDF CH_2_ wagging vibration. The band at 1180 cm^−1^ represents the asymmetric stretching vibration of the CF_2_ group and the 1068 cm^−1^ band is attributed to the CH_2_ wagging mode; β phase band at 874 cm^−1^ is related to the CF_2_ symmetric stretching; the α-phase characterization band at 763 cm^−1^ and also at 610 cm^−^^1^ belong to the CF_2_ bending [66,67]. The absorption peaks at 880 and 841 cm^−1^ correspond to C-C-C and CF stretching vibration of PVDF [66]. The exclusive peaks for the piezoelectric β phase are observed around 445 cm^−^^1^, 473 cm^−^^1^, and 1275 cm^−^^1^ [68]. The chemical structure of the prepared nanocomposite membrane showed the characteristic absorption peaks of pure PVDF. The absorption peak at around 1662 cm^−^^1^ which is reported to correspond to the addition of silver [69] did not occur, probably due to the low AgNPs concentrations, but Ag presence was confirmed with EDS.

The peaks at 840 cm^−^^1^ and 510 cm^−^^1^ describe the PVDF characteristics in a specific way. In the case when the peak at 1275 cm^−1^ is present, but with the absence of the 1234 cm^−1^, then the 840 cm^−1^ and 510 cm^−1^ bands correspond to the β phase. Furthermore, when the peak at 1275 cm^−1^ is absent and with the presence of a peak at 1234 cm^−1^, the 840 cm^−^^1^ and 510 cm^−^^1^ bands correspond to the γ phase. In the case when both 1275 cm^−1^ and 1234 cm^−^^1^ are present, the two former bands are considered as both the β and γ phases [68]. Accordingly, absence of the 1234 cm^−^^1^ band indicates that 510 cm^−1^ and 840 cm^−1^ bands can be considered as the β phase.

The relative fraction of the electroactive phases (β and γ) can be quantified according to the Lambert–Beer law [68,70]:

(1) $$F_{\beta }=\frac{A_{\beta }}{\left(\frac{K_{\beta }}{K_{\alpha }}\right)A_{\alpha }+A_{\beta }}\times 100$$

where $A_{\alpha }$ and $A_{\beta }$ are the absorbencies at 763 cm^−1^ and 840 cm^−1^, respectively; $K_{\alpha }$ and $K_{\beta }$ are the absorption coefficients at the respective wave numbers, obtained from [68,71]. The calculated fraction of the β phase in our samples is presented in Table 2.

The FTIR results indicate an increase in the β phase presence in the electrospun fibers as compared to the solution casted samples, which can be explained by the high voltage applied to the polymer solution and the large mechanical elongation of the fluid jets during the electrospinning process [68,72]. In addition, incorporating AgNPs into PVDF nanofibers resulted in a further β-phase increase with concentrations of 0.1% and 0.3% AgNPs, whereas the β-phase reduction was noticed for the AgNPs increase to 0.5%, as also reported in the literature for the higher AgNPs concentration [73].

### 3.2. In Vitro Cytotoxicity Assay

Evaluation of the cytotoxicity of the PVDF nanofibers was carried out on two different cell lines: healthy fibroblast MRC-5 and MDA-MB-231 breast cancer cell lines. The selection of the MDA-MB-231 cell line was based on the previous literature data indicating an increased susceptibility of this cell type to the effects of silver [74,75,76].

**Figure 7:**
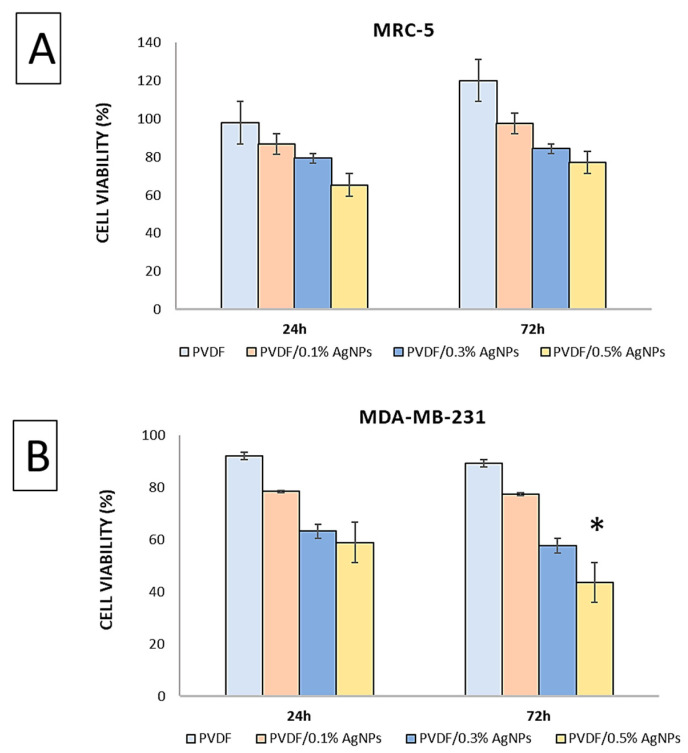
Cytotoxic effect against (**A**) MRC-5 and (**B**) MDA-MB-231 cell lines. * Represents statistical significance in comparison with a pure PVDF sample at *p* < 0.05.

Figure 7 presents the results of the assessment of the viability of MRC-5 cells after 24 h and 72 h. The analysis revealed the percentage distribution of the cell viability, where the PVDF showed a viability of 97.3 ± 0.82 after 24 h, and PVDF/0.1% AgNPs, PVDF/0.3% AgNPs, and PVDF/0.5% AgNPs showed viabilities of 86.78 ± 1.35, 79.34 ± 1.96, and 65.26 ± 3.12, respectively. Subsequent evaluation after 72 h showed the following increase in the percentage of cell viability: 120.12 ± 3.87, 97.46 ± 4.19, 84.38 ± 1.48, and 77.02 ± 1.24, respectively. The results significantly suggested that both the pure PVDF and the PVDF/AgNPs nanofibers did not show cytotoxic effects on the healthy cells during the experimental period, with no significant differences observed between the samples. However, a comparison of the results revealed that pure PVDF showed a higher viability after both 24 h and 72 h as compared to the PVDF/AgNPs nanofibers. Furthermore, as the concentration of Ag increased in the PVDF nanofibers, the cell viability showed a corresponding decrease, as shown in Figure 7.

In contrast, findings regarding MDA-MB-231 cells illustrate a decrease in cell viability with increasing AgNPs content in the PVDF nanofibers. After 24 h, the viability percentages were 92.09 ± 6.25 for pure PVDF, 78.43 ± 1.23 for PVDF/0.1% AgNPs, 63.21 ± 1.95 for PVDF/0.3% AgNPs, and 58.92 ± 2.93 for PVDF/0.5% AgNPs. After 72 h, the percentages were 89.34 ± 8.10, 77.43 ± 5.71, 57.67 ± 3.48, and 43.54 ± 6.64, respectively. Significantly, the PVDF/0.5% AgNPs nanofibers showed the most pronounced cytotoxic effect against MDA-MB-231 breast cancer cells. It is important to note that the IC_50_ value was 0.4% for the PVDF/AgNPs nanofibers after 72 h.

#### Analysis of the Antibacterial Activity of PVDF/AgNPs Fiber Mats

**Figure 8:**
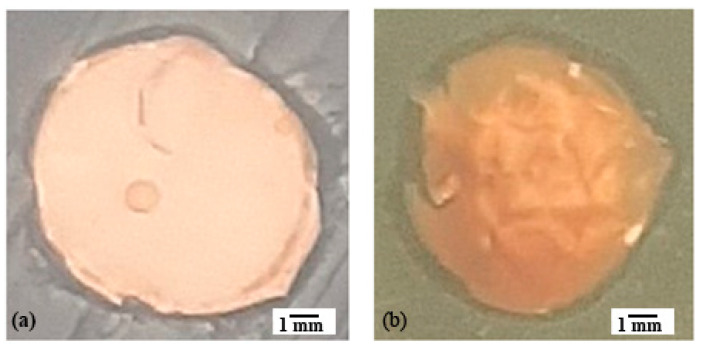
An example of the action of PVDF/AgNPs (0.3%) nanofiber on (**a**) *Staphylococcus aureus* ATCC 25923, (**b**) *Pseudomonas aeruginosa* ATCC 27853.

**Figure 9:**
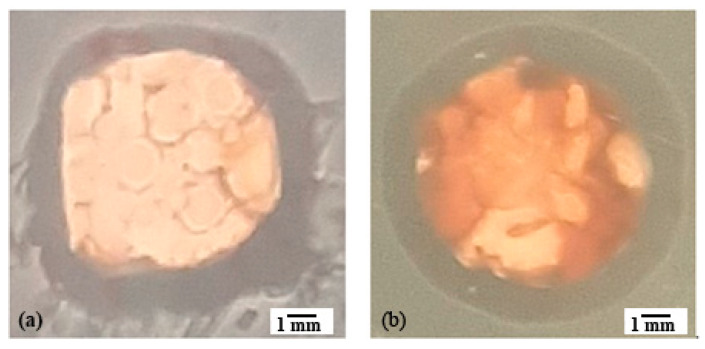
An example of the action of PVDF/AgNPs (0.5%) nanofiber on (**a**) *Staphylococcus aureus* ATCC 25923, (**b**) *Pseudomonas aeruginosa* ATCC 27853.

Antimicrobial activity was determined for the Gram-positive bacteria *Staphylococcus aureus* ATCC 25923 and the Gram-negative bacteria *Pseudomonas aeruginosa* ATCC 27853, by the disc diffusion method. The results showed that the PVDF nanofibers with different AgNPs concentrations have a certain degree of antimicrobial activity that increases with increasing the AgNPs concentration (Table 3). The diameters of the inhibition zones of the PVDF nanofibers with 0.3% and 0.5% AgNPs indicates the potential antimicrobial activity of the material, especially at the point of application (Figure 8 and Figure 9).

## 4. Discussion

The morphology of PVDF and PVDF/AgNPs exhibits a distinct tendency. The pure electrospun PVDF nanofibers without silver have a morphology that involves interfiber bead formations. This phenomenon is primarily caused by the insufficient polymer solution validation, which can be attributed to the factors such as the absence of the solution conductivity, inappropriate viscosity, or deviations in the environmental parameters. The incorporation of silver has a significant impact on the morphology of PVDF nanofibers, as shown in Figure 2. The addition of the AgNO_3_ salt increases the level of the electrical charge of the polymer solution and the elongation capacity of the solution jet during the electrospinning process, thus promoting the formation of smoother fibers with smaller diameters and higher uniformity [77,78]. There is a significant reduction in the beads and improvement in fiber uniformity, even at low concentrations (PVDF/0.1%AgNPs). It can be noticed from the SEM micrographs that the morphology improves with an increase in the silver percentage.

The longest diameter (569.3 ± 216.9 nm) was exhibited in the case of the PVDF/0.3% AgNPs nanofibers. This could be related to the appearance of aggregation during the electrospinning process, when the polymer jet is stretched until longer diameters are produced. Otherwise, that increase could be attributed to the AgNPs agglomerates inside the nanofibers [79]. The nanofiber diameter is an essential parameter for various applications, including interactions with the physiological environment [63]. In our article, the anticancer, antimicrobial, and piezoelectric properties are highlighted. Nanofiber diameter can have a significant impact on the interactions between the cells and the surrounding materials. A shorter diameter of the nanofibers can efficiently mimic the extracellular matrix (ECM) structure, thus promoting better adhesion and interactions with cancer cells. The shorter diameter of the nanofibers exhibits a higher surface area to volume ratio, meaning that a higher contact area between the nanofibers and microorganisms is available, which further improves the effectiveness of the antimicrobial agents such as AgNPs. The morphological and mechanical properties of the resulting nanofibers influence their interactions with microbes. The uniform nanofibers maintain the structural integrity of the bulk nanomaterial and effectively perform interactions during antimicrobial treatments [80]. In piezoelectric materials that produce an electrical charge in response to mechanical stress, the diameter of the nanofibers is directly correlated with the density of the piezoelectric domains within the material. A shorter diameter of the nanofibers can improve piezoelectric properties by increasing the crystalline domain density or enable better orientation of the piezoelectric dipoles [81].

The impurities can have an impact on the preparation and evaluation process. Contamination is low during the electrospinning process because the chamber of the electrospinning system is isolated. The highest risk of contamination may occur during the sample evaluation. In the case of vitro experiments, such as cytotoxicity testing and microbiology evaluation, it is essential to reduce contamination during the sample handling. During the cell and bacterial experiments, sterile conditions must be maintained. Samples might be contaminated during the gold coating for the SEM analysis. The sample preparation for FTIR analysis was performed under sterile conditions since the contaminants can reduce the purity and performance of the PVDF nanomats. The impurities in nanomats can alter their properties, provoke some undesirable reactions (e.g. increased cytotoxicity), and limit their suitability for biomedical or environmental applications.

Comprehensive insight into the chemical composition and elemental distribution of the incorporated agents was achieved by combining the FTIR and EDS methods. The EDS method was used to identify AgNPs on the surface of PVDF nanofibers and provide the elemental distribution. With an increase in the AgNPs percentage, from 0.1% to 0.5%, nanoparticles are better integrated into the PVDF nanofibers. Despite the presence of the AgNPs, the distribution of the F atom in each of the samples is mainly unaffected, thus suggesting that the PVDF chemical structure is preserved. The impact of the incorporated AgNPs agents contributed to the understanding of the elemental distribution and composition of the polymeric PVDF nanofibers. The presence of C, O, N, and F atoms confirmed the chemical composition of the PVDF nanofiber structure. Silver nanoparticles have been successfully integrated into the PVDF nanofibers in all three samples, as can be seen in Figure 4.

The homogeneous distribution of AgNPs within the PVDF electrospun nanofibers has a major influence on the electrospinning process. The uniformity and morphological properties of the fabricated nanofibers strongly depend on the adequate electrical conductivity of the starting polymer solution where a homogeneous distribution of the AgNPs strongly enhances its electrical conductivity. In the case of applications where strong antimicrobial activity is required, the presence of larger quantities of silver might be necessary, such as the samples with 0.5% of AgNPS in PVDF nanofibers in our research. Also, with higher electrical conductivity as the main objective of the designed smart material, higher silver concentrations might be needed. However, in our cytotoxicity tests, it was important to optimize the silver concentration to produce the optimal balance between its effects on healthy and cancer cells. A homogeneous distribution of the AgNPs ensures consistent and improved properties throughout the nanomaterial, while the volume fraction of the added AgNPs should be tailored to the specific end-use requirements and considering the desired functional improvements.

Other authors have studied the addition of AgNPs [52] or hybrid AgNP/MXene (transition metal carbide/nitride) nanoparticles [82] to PVDF and fabricated piezoelectric composite nanofibers by near-field electrospinning. They also concluded that both pure AgNPs and AgNPs with MXene nanoparticles significantly enhanced piezoelectric properties (increased piezoelectric constant and electrical conductivity), which is in accordance with our research. They concluded that the AgNPs with MXene increased the electrical conductivity of the hybrid PVDF nanocomposite from 40 µS/cm to 1148 µS/cm [82], while in the case of pure AgNPs, there was an increase up to 883.59 µS/cm [52]. They reported approx. 80% of the β-phase content, with the AgNPs added to the PVDF, which is comparable to our results for pure PVDF. It is a slightly lower value than for our samples with the AgNPs (where we calculated approx. 91–96% for 0.1–0.3% AgNPs). However, they did not vary the AgNPs content, whereas we obtained a β-phase decrease with a further increase in the silver percentage. They used 10 wt% of the AgNPs relative to MXene wt% [82] and in another study 10% of AgNPs [52], but such a high silver concentration is harmful for the healthy cells, as we showed in our research. They also showed distinctive FTIR peaks of the Ag crystals as in our research.

Selection of an adequate cosolvent (DMF) for the AgNPs addition has been proven by the presence of the β phase in FTIR spectra (Figure 6). The DMF cosolvent promotes the formation of the –CH_2_/–CF_2_ dipole and enhances the β phase in PVDF, whereas the solution-casted film exhibited 46% of the β phase, as shown in Table 2. Afterwards, when that same solution was subjected to a strong electrical field and stretching during the electrospinning process, more dipole formation was initiated and resulted in 80% of the β-phase content in pure PVDF electrospun nanofibers [68,72]. Finally, with the addition of the AgNPs, the β-phase fraction increased to 91% and 96% for 0.1% AgNPs and 0.3% AgNPs, respectively. However, a further increase in the silver concentration resulted in a β-phase reduction, which is in agreement with other published research [73].

The anticancer properties of the AgNPs have been shown in relation to the breast cancer cell lines [83,84]. Accordingly, the MDA-MB-231 breast cancer cell line and the healthy MRC-5 cell line were used in our investigation. The cytotoxicity and biocompatibility of the pure PVDF, PVDF/0.1% AgNPs, PVDF/0.3% AgNPs, and PVDF/0.5% AgNPs nanofibers were evaluated by using the MTT test after a 24 h and 72 h incubation period. The cell viability tests for each of the samples demonstrated non-toxic effects, as shown in Figure 7. It should be noted that there was a dose-dependent increase in the cell viability. Accordingly, after 72 h, the cell viability on MRC-5 cells was the highest for pure PVDF (120.12 ± 3.87) and the lowest for PVDF/0.5% AgNPs samples, which is an acceptable compatibility for biomedical applications.

In contrast, the breast cancer cell line MDA-MB-231 showed a cytotoxic response that also followed a dose-dependent trend, particularly pronounced after a longer incubation period (Figure 7). The cytotoxic effects increased after 72 h, with cell viability significantly decreasing with the increase in the AgNPs concentration. The most significant decrease in viability was observed for the PVDF/0.5% AgNPs sample, thus indicating statistically significant cytotoxic effects as compared to the pure PVDF sample (IC_50(72h)_ = 0.4%). This outcome can be attributed to the interaction between negatively charged cancer cell membranes and the remaining positively charged Ag^+^ ions that are released from the PVDF/Ag composite, leading to a disruption of cellular metabolic activities and subsequent cell death [85].

It can be elaborated that the underlying mechanism has involved the generated reactive oxygen species (ROS), which is also supported by other published research. The interaction between the oxygen and hydrogen atoms in the presence of the Ag^+^ ions increases the ROS production, with its negative effect on the pathological state of the cancer cells. Increased ROS levels can cause oxidative stress that further results in the structural modification of the cells and reduced cell proliferation [86].

It is known that PVDF/PVP/AgNPs electrospun membranes exhibit antibacterial activity by forming an inhibition zone around the edges of the membrane samples against *Staphylococcus aureus*, which is also shown in our research. The increased concentration of the silver nanoparticles (from 1 to 2.5 wt.%) increased the antibacterial activity, as shown by the inhibition zones around the samples [87,88], which is in accordance with our research. The research of other authors has also indicated 3–4 mm inhibitory zones and antibacterial properties against Gram-negative bacteria in the case of PA/PVDF composites with AgNPs (with sizes of 20 nm to 50 nm) [65], which is similar to our results.

The essential process parameter for a successful fabrication of nanofibers in electrospinning is electrical conductivity, whereas the AgNPs can increase the electrical conductivity of the polymer solution. Electrical conductivity is needed in applications such as sensors and flexible electronics. The AgNPs incorporated into the fibers may provide antimicrobial properties, which is suitable for applications such as medical textiles, wound dressings, and air filtration systems. Also, our study showed the anticancer properties of the PVDF/AgNPs. Silver is widely regarded as biocompatible and nontoxic and can be used in medical and consumer products when properly stabilized. Accordingly, the AgNPs can be incorporated into fibers intended for biomedical applications with minimal toxicity concerns. The disadvantages can include the high cost, possible incompatibility with a specific polymer, possible harmful effects for higher concentrations, and the agglomeration of silver particles due to their higher concentration.

Conducting property studies related to variation in AgNPs sizes would be worthwhile, especially considering its influence on breast cancer cells. Here, the presented combination in breast cancer treatment needs further studies on composites with different polymers, as also indicated by our previous research related to another type of polymer and cytotoxicity on the MRC-5 cell line [62]. The PVDF electrospun nanofiber samples can be additionally functionalized for their antibacterial activity, for example by using natural polymers with reported antimicrobial properties, such as gelatin or chitosan. The blend combination of synthetic PVDF and natural polymer can improve the antibacterial activity of nanomats in combination with different antimicrobial nanoparticles. Also, bioactive molecules such as antibiotics or antimicrobial peptides can be loaded onto PVDF nanofibers to provide sustained antibacterial activity. Apart from cancer treatment studies, the custom design of material microstructures with nanoparticles distributed throughout the nanofiber-based composites with functional properties such as piezoelectricity can effectively serve in the novel design of hierarchical nanobiosensors for virus detection [89]. The emerging concept of nanoarchitectonics [90], which integrate existing research at nano-scales, including a reproduction of cellular functions or hybrid nanostructures with hierarchy, in combination with the custom design of composite properties, enabled by additive technologies, should be further studied aiming at functional materials that can help in cancer treatment and combating viruses.

It is proven that modifications by laser light can enhance piezoelectricity, like in crystalline [91] and lead-based piezoelectric materials [92]. However, pure PVDF has photo-inactive polymer chains, even though some new photolithographic additive technologies have been studied with modified resin compositions to provide photoactivity of the PVDF-based piezoelectric materials [93,94], thus indicating that the influence of intensive light should be further investigated. We did not study the influence of intensive light on the piezoelectric properties of PVDF-based composites in our research, but results from other authors, such as in the case of graphene oxide-doped PVDF [95], indicated that the addition of AgNPs in our samples might make them sensitive to the light intensity from aspects of structural phases, which should be further investigated for practical applications. Piezo-phototronic effects, or coupled effects between the piezoelectricity, photonic properties, and semiconductors, are still not well understood, even though these phenomena represent the foundation for a range of novel optoelectronic nanodevices that have shown exquisite possibilities to tailor and enhance performance efficiency in different potential biomedical applications, including chemical and biological optoelectronic nanoscale devices [96].

The great importance of PVDF-based piezoelectric materials for use in biomedical sensors, self-powered medical devices, advanced smart biomedical implants, flexible wearables, and energy harvesters, including novel 4D smart systems, to list a few possible applications, drives and justifies the research on such materials’ design.

## 5. Conclusions

In this study, PVDF and PVDF/AgNPs composite nanofibers were fabricated by the electrospinning process. A pure PVDF sample was also prepared via the solution-casting method to compare the influence of the electrospinning process on the content of the crystalline β phase. EDS demonstrated the existence and homogeneous distribution of AgNPs, confirming the structural integrity and its successful binding to the PVDF nanofibers. FTIR characterization was used to determine the β-phase fraction in the composite nanofibers. It was noted that the electrospinning process increases the β-phase fraction (80%) compared to the solution-casted sample (46%), and further increased with the addition of 0.1% and 0.3% AgNPs leading to 91% and 96%, respectively, while the addition of 0.5% AgNPs resulted in a β-phase reduction to 60%. It can be concluded that AgNPs enhanced the piezoelectric properties of the PVDF nanomats. Furthermore, it was concluded that the composite nanofibers with AgNPs exhibited a higher conductivity of the polymer solution, with uniform morphology, and shorter diameters of the produced nanofibers. Also, the PVDF/0.5% AgNPs nanofibers showed a prominent effect on the MDA-MB-231 breast cancer cell line (IC_50(72h)_ = 0.4%) and non-toxicity on the healthy MRC-5 cell line. The PVDF/AgNPs nanofibers showed promising antibacterial activity against *S. aureus* and *P. aeruginosa* at the Ag contents of 0.3% and 0.5% Ag.

Our research showed that piezoelectric PVDF/AgNPs nanomats can be potentially used as multifunctional materials in cancer treatment, and specifically target breast cancer cells, thus presenting significant further potential research impacts. However, further research is needed to reveal the effects of AgNPs sizes, processing parameters and interdependence between the material properties, and applied voltage during electrospinning. Different combinations of polymers and possible functionalization to improve bioactivity should be investigated related to breast cancer treatment.

Incorporation of AgNPs into PVDF nanofibers may enable wider applications of such piezoelectric materials for medical textiles, wound dressings, and air filtration systems with antibacterial effects. Sustainable, lead-free, PVDF-based piezoelectric materials have enormous potential for use in biomedical sensors, self-powered medical devices, advanced smart biomedical implants, flexible wearables, and energy harvesters, including novel 4D smart systems.

Some recent research related to piezo-phototronic effects has indicated possible ways to control and enhance the energy conversion efficiency of these materials, by using a laser-induced intensive light to increase electric conductivity and produce a better stability, mechanical strength, and sensitivity of the polymer piezoelectric materials, aiming at advanced optoelectronic nanodevices that can enhance performance efficiency. Further research should enable a better understanding of the underlining phenomena in the coupling effects of mechanical, piezoelectric, and semiconductor properties in polymer-based composites and especially related to different optoelectronic nanoscale devices in biomedical applications and wider chemical and biological biosensors.

Custom-designed functional nanocomposites also have a very significant potential for antiviral treatment; hence, the design of such hierarchical nanobiosensors is a promising research area related to pandemic prevention. The emerging concept of nanoarchitectonics to integrate different aspects in design of materials at nanoscales, in combination with novel additive technologies, opens new research directions on functional materials that can help in cancer treatment.

## Figures and Tables

**Table 1:** The best solution and parameters of the electrospinning.

Solution		Electrospinning Parameters				
PVDF Concentration [%]	Solvent [*v*/*v*%]	Voltage [kV]	Needle [gauge]	Flow Rate [mL/h]	Tip to Collector Distance [cm]	Temperature, Humidity
21%	75% DMF:25% Ac	30	18	0.5	15	30 °C, 45%

**Table 2:** The beta phase fraction in different samples.

Sample	$F_{\beta }$ [%]
solution cast	46%
Pure PVDF	80%
PVDF/0.1% AgNPs	91%
PVDF/0.3% AgNPs	96%
PVDF/0.5% AgNPs	60%

**Table 3:** Zone diameter results of the tested material by the disk diffusion method.

Tested Bacteria/Tested Material	*Staphylococcus aureus* ATCC 25923 [mm] *	*Pseudomonas aeruginosa* ATCC 27853 [mm] *
PVDF/AgNPs (0.1%) nanofiber	-	-
PVDF/AgNPs (0.3%) nanofiber	7 ± 0.00	8 ± 0.47
PVDF/AgNPs (0.5%) nanofiber	10 ± 1.25	10 ± 0.47
Gentamicin tests disc 10 μg	21 ± 1.25	19 ± 0.82

* The inhibition zone diameter is given in mm with the surface of the tested material. - no inhibition zone.

## Data Availability

The authors declare that all data supporting the findings are available within the paper or are available from the authors upon request.
